# Source for In Situ Positron Annihilation Spectroscopy of Thermal—And Hydrogen-Induced Defects Based on the Cu-64 Isotope

**DOI:** 10.3390/ma14216693

**Published:** 2021-11-06

**Authors:** Iurii Bordulev, Roman Laptev, Denis Kabanov, Ivan Ushakov, Viktor Kudiiarov, Andrey Lider

**Affiliations:** 1 Division for Experimental Physics, National Research Tomsk Polytechnic University, 634050 Tomsk, Russia; laptevrs@tpu.ru (R.L.); kudiyarov@tpu.ru (V.K.); lider@tpu.ru (A.L.); 2 Research Nuclear Reactor Center, National Research Tomsk Polytechnic University, 634050 Tomsk, Russia; kabanoff@tpu.ru (D.K.); jiaozu@tpu.ru (I.U.)

**Keywords:** positron annihilation, defects, in situ, neutron activation, ^64^Cu, nuclear reactor, hydrogen-induced defects, thermal vacancies

## Abstract

This work aims to investigate the ^64^Cu isotope applicability for positron annihilation experiments in in situ mode. We determined appropriate characteristics of this isotope for defect studies and implemented them under aggressive conditions (i.e., elevated temperature, hydrogen environment) in situ to determine the sensitivity of this approach to thermal vacancies and hydrogen-induced defects investigation. Titanium samples were used as test materials. The source was obtained by the activation of copper foil in the thermal neutron flux of a research nuclear reactor. Main spectrometric characteristics (e.g., the total number of counts, fraction of good signals, peak-to-noise ratio) of this source, as well as line-shaped parameters of the Doppler broadening spectrum (DBS), were studied experimentally. These characteristics for ^64^Cu (in contrast to positron sources with longer half-life) were shown to vary strongly with time, owing to the rapidly changing activity. These changes are predictable and should be considered in the analysis of experimental data to reveal information about the defect structure. The investigation of samples with a controlled density of defects revealed the suitability of ^64^Cu positron source with an activity of 2–40 MBq for defects studies by DBS. However, greater isotope activity could also be applied. The results of testing this source at high temperatures and in hydrogen atmosphere showed its suitability to thermal vacancies and hydrogen-induced defects studies in situ. The greatest changes in the defect structure of titanium alloy during high-temperature hydrogen saturation occurred at the cooling stage, when the formation of hydrides began, and were associated with an increase in the dislocation density.

## 1. Introduction

The study of defects evolution in structural and functional materials under aggressive operating conditions is an urgent task for modern material science. This work aims to investigate the use of the ^64^Cu isotope for in situ studies of the defect structure evolution of these materials under aggressive conditions, such as high temperatures and hydrogen saturation.

The problem of hydrogen interaction with metals and alloys is not new [1,2]. Modern studies of metal-hydrogen systems are motivated by fundamental questions of the hydrogen dissolution effect on the major physical and chemical properties of materials. In addition, the use of functional and structural materials interacting with hydrogen under various conditions are studied for fields such as hydrogen energy, petrochemical, automotive, aerospace, and nuclear industries. The interactions of solids with hydrogen are accompanied by processes such as the new phase formation and the development and evolution of hydrogen-induced defects (vacancies, dislocations, vacancy clusters, and “defect-hydrogen” complexes [3,4,5,6]). Changes in the defect structure affect essential material characteristics, including mechanical, electrical, and sorption properties. Thus, to create novel, promising materials in the above-mentioned industries, the investigation of the evolution of the defect structure under aggressive operating conditions (in particular at elevated temperatures and in a hydrogen environment) in situ is needed.

A sensitive method to study open-volume defects is positron annihilation spectroscopy (with a sensitivity of 10^−7^ vacancies/atom [7]). This method has been successfully used to study basic defects, including vacancies, dislocations, vacancy clusters [8,9,10], and complex defect structures, such as hydrogen-induced defects [3,11,12,13]. The studies on defect structures were conducted with hydrogen loading from an ex-situ saturation process. However, the in situ investigation of the defect structure evolution during hydrogen exposure is more useful in terms of the mechanisms of hydrogen embrittlement. Experiments on the hydrogen interaction with materials are carried out either using the liquid phase (electrolytic saturation) [14] or gas phase (hydrogen gas sorption at high pressure and various temperatures) [15]. These conditions impose more stringent requirements for the potential positron sources to be used for in situ studies.

The positron sources currently used in positron annihilation methods are divided into two large groups: radioisotope sources and high-intensity positron beams at acceleration complexes. The first group includes various β^+^ isotopes, the most popular of which is ^22^Na. A source based on ^44^Ti is produced in the Russian Federation. Positron sources based on isotopes are radioactive substances (often in the form of salts) packed in a protective capsule with an exit window. Mylar or Kapton (for ^22^Na [16]) and pure titanium (for ^44^Ti [17]) are used as the capsule material. These sources cannot be used for in situ studies, as the capsule materials are not designed for operation under aggressive conditions, such as exposure to acid solutions, high temperatures, or the presence of hydrogen. Damage to the capsule can create conditions for leakage of the source and radioactive contamination of the environment. Work has been performed on obtaining positron sources directly inside the materials under study. Thus, using nuclear reactions, “internal” sources of positrons based on isotopes, such as ^22^Na, ^58^Co, ^64^Cu, and ^44^Ti, were obtained [18,19]. Although these sources can be used for in situ studies, the main disadvantage of this approach is the very limited choice of materials. The use of intense positron beams also encounters difficulties with its application to in situ studies of material—hydrogen interaction. These difficulties, adding to the costliness of the method itself, are related to the beam delivery to the studied material since the manipulations with the positron beam are performed in a vacuum, while the studied material is surrounded by a medium (either gas under pressure or liquid).

Thus, no experimental technique is available for the study of hydrogen-induced defects in situ during the saturation process. The main purpose of this paper is to fill this gap.

This paper proposes the use of a positron source based on the ^64^Cu isotope. This source can be obtained using the ^63^Cu (n, γ) ^64^Cu reaction by irradiating a copper foil with a flux of thermal neutrons. Pure copper, with a high melting point, very weakly interacts with hydrogen compared to other materials [20,21,22,23,24,25,26,27,28], which allows it to be used in a heated hydrogen environment under high pressure.

A typical feature of this positron source is its short half-life (12.7 h). On the one hand, this solves the issues of radiation safety and the disposal of the used source. On the other hand, since changes in the activity of the positron source affect some characteristics of the positron annihilation spectra, rapid changes in these parameters will need to be considered in future experiments. Another advantage of the ^64^Cu source is the simplicity of its design. Since the material is just a metallic foil, the activity of which quickly decays in a few days, this source does not require a protective capsule. A disadvantage of this isotope is the limitation of its application to momentum distribution techniques, such as the angular correlation of annihilation radiation (ACAR) and Doppler broadening spectroscopy (DBS). The use of ^64^Cu for positron annihilation lifetime spectroscopy is not possible because of the lack of a starting photon in the reaction. This source has already been used in in situ studies of the metals’ defect structures by the ACAR method during heating [29,30]. However, applying this isotope in DBS has not yet been investigated. Thus, this novel study explores the effect of ^64^Cu in the DBS technique and assesses hydrogen-induced defect formation in situ using this isotope.

## 2. Materials and Methods

### 2.1. Positron Source

The positron yield for the ^64^Cu isotope is ~18% [31], which is lower than that of standard positron sources (such as ^22^Na and ^44^Ti). Thus, for the efficient use of a ^64^Cu source, higher activities (>3 MBq) than those in standard positron sources are necessary.

The source material was copper foil (purity 99.99%) with a thickness of 10 µm and a mass of 1 mg. In general, to improve the sensitivity of the experiment, the authors used the thinnest possible foil to increase the fraction of positrons annihilating in the material under study. Since the end-point energy of positrons emitted by ^64^Cu is 0.657 MeV, the source contribution using this foil would be at least 45% (without considering backscattering). To remove surface impurities and dissolved gases, the foil was etched in a 2M HCl solution and annealed in a vacuum at 700 °C for 3 h.

The vertical dry experimental channel of Tomsk Polytechnic University Research Nuclear Reactor (IRT-T) was used in this work to irradiate the copper foil. The average thermal neutron flux in this channel was around 10^13^ n⋅s/cm^2^. Irradiation was carried out for 3 h followed by exposure outside the neutron field for 2 h. The activity at the beginning of the experiment was ~40 MBq.

### 2.2. Samples

The positron source was placed between two identical test samples of 2-mm thick titanium in a “sandwich geometry.” The samples were annealed in a vacuum at 790 °C for 1 h. To obtain samples with different levels of defects, one sample set was deformed up to 1% from the original thickness, and the other was deformed 10% by cold rolling.

### 2.3. Heating and Hydrogen-Loading Experiments

Each measurement was carried out in a steel vacuum chamber located in a high-temperature furnace. The vacuum (of around 1.3 × 10^−5^ bar) and hydrogen atmosphere were created with the Gas Reaction Controller equipment [32].

To measure positron annihilation characteristics at high temperatures in situ, samples with a ^64^Cu source were heated from room temperature (around 20 °C) up to 773 °C in four steps. Heating between steps was carried out for 1 h, while the exposure at a constant temperature (at each step: approximately 190, 400, 590, 770 °C) was performed for 3 h. After soaking at the maximum temperature, samples were cooled to room temperature also in four steps. In each cooling step, the samples were kept at a constant temperature for 3 h.

Hydrogen loading was performed at the same temperature mode as the vacuum heating with hydrogen pre-injection in the chamber at room temperature. The initial hydrogen pressure was set to 1.8 bar and was not maintained at a constant level during measurement. All changes in hydrogen pressure were due to heating and hydrogen absorption by the samples.

For each measurement, new copper foil was prepared.

The absolute hydrogen concentration in the samples and foil was measured by melting the sample in an inert gas medium using the RHEN LECO facility [33].

### 2.4. Doppler Broadening Spectrometer

The DBS method of positron spectroscopy was used in this work [7] and is based on the measurement of the energy shift resulting from the non-zero pulse of the electron involved in the annihilation process. This method, along with its high sensitivity to changes in the defect structure of materials, is also quite rapid in comparison with other methods of positron annihilation, based on the measurement of positron lifetime and angular distribution of annihilation *γ*-quanta.

For this experiment, the authors used the spectrometer described in [6]. Its main part consists of a detector based on high-purity germanium (HPGe) and a high-speed digitizer. Two different HPGe detectors were used for the “in-air” experiment (virgin sample and deformed samples) and the experiment in a vacuum (virgin sample at room temperature, at high temperatures, and during hydrogen loading). Therefore, some characteristics varied for different spectrometers (see Table 1).

**Figure 1:**
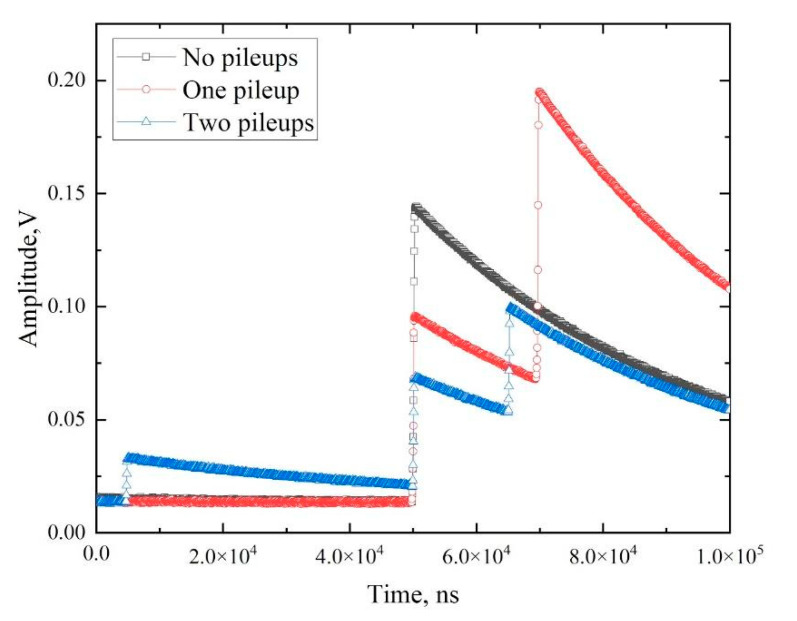
Signal waveforms from the HPGe detector of Doppler broadening spectrometer with different numbers of signal pileups.

The “bad” events were filtered out at the level of digital signal processing. The appearance of digitized signals from the preamplifier Ge detector of the DBS spectrometer is shown in Figure 1.

As shown in Figure 1, the signals from the preamplifier can be single and overlapped. The latter case occurs when a detector registers two or more particles during one collection. Such a pileup of signals complicates their further processing, particularly in the estimation of the amplitude and, thus, the photon energy. The software used in the spectrometric module (developed by the Positron Annihilation Group of Charles University, Prague) [34] successfully processes oscillograms without overlapping as well as with one pileup. In the case of three or more overlapping signals, the oscillogram is classified as “bad” and cannot be further processed.

The specialized software SP was used to analyze the DBS spectra [35]. The Doppler broadened 511-keV peak is the electron momentum distribution convoluted with the detector resolution. The analysis primarily involves the estimation of the spectrum line-shape parameters (*S*- and *W*-parameters). The *S*-parameter is the ratio of the spectral area in the locality around the 511 keV peak to the total peak area, and the *W*-parameter is the ratio of the area of two symmetrically located intervals on the sides of the 511-keV peak to the total area. Usually, the energy ranges used to calculate *S*- and *W*-parameters are determined such that the values of the *S*- and *W*-parameters are close to 0.5 and 0.03, respectively [7]. Changes in *S*- and *W*-parameters indicate changes in the probability of positron annihilation with valence and core electrons, respectively. In terms of the effect on the DBS spectra, the capture of positrons by defects leads to an increase in the *S*-parameter and a decrease in the *W*-parameter. In this case, the spectrum, in which the concentration of defects is higher, becomes visibly narrower. This phenomenon is caused by the increase in the fraction of valence electrons taking part in the annihilation process, in the case of positron localization in open-volume defects, compared with that of core electrons [7].

A total of 20 × 10^6^ signals (bad and good) were registered and digitized in each measurement, which took from 150 to 470 min per measurement.

## 3. Results and Discussion

### 3.1. Performance of ^64^Cu at Room Temperature

The analysis of the spectrometric characteristics of the obtained source includes a study of correct signal fraction, number of counts, peak-to-noise ratio, and line-shaped parameters (*S* and *W*) of the annihilation line at different isotope activity levels.

**Figure 2:**
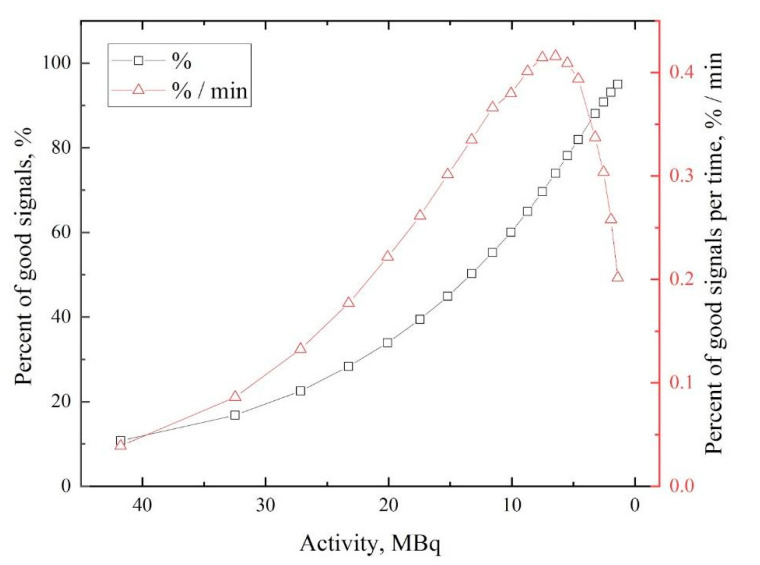
Dependence of “good” signals percentage (absolute and related to time values) on the ^64^Cu activity.

The dependence of the “good” events fraction on the activity of the used isotope is presented in Figure 2.

**Figure 3:**
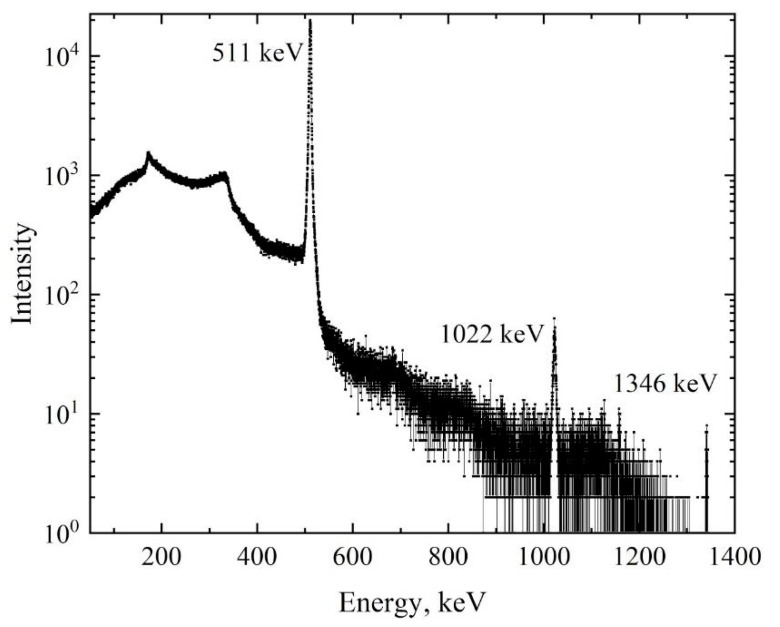
Energy *γ*-radiation spectrum of ^64^Cu isotope.

Figure 2 shows that the fraction of “good” signals increases with time (with activity fall). The number of “good” events improves with a slower registered signals count rate (since registering two or more signals at one time window will lead to a pileup) and depends on the ratio of detector deadtime, which is typically inversely proportional to the number of signals per time. However, the time needed for one spectrum collection also increases with time. Taking this into account, the dependence of the number of “good” signals related to the time of spectrum collection on isotope activity is also determined from Figure 2. The maximum value of this function indicates the highest effectiveness of the acquisition process. According to this dependence, the most appropriate activity value of ^64^Cu isotope for the positron annihilation experiment is in the range of 5–8 MBq. The energy spectrum of *γ*-radiation of the obtained ^64^Cu isotope is shown in Figure 3.

**Figure 4:**
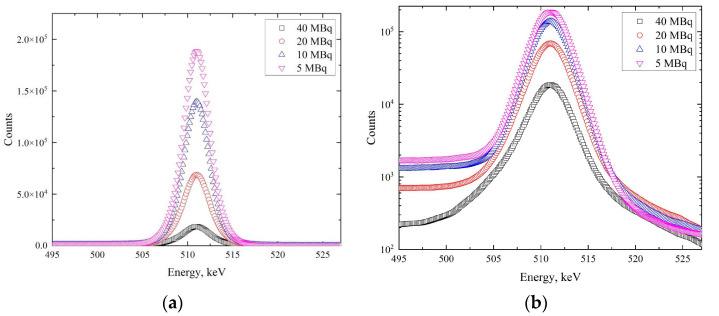
Energy spectra of annihilation line at the different activity levels of the ^64^Cu isotope: linear scale (**a**), logarithmic scale (**b**).

The presented distribution shows intense peaks of 511 keV (positron annihilation emission), 1022 keV (the double absorption of annihilation photons), and 1346 keV (emission arising from the transition of ^64^Ni from an excited state to the ground state). In the context of the DBS method, only one line, which corresponds to the energy of 511 keV, is analyzed. The energy spectra of the *γ*-radiation in the region of the 511 keV peak for different activities of the positron source are presented in Figure 4.

**Figure 5:**
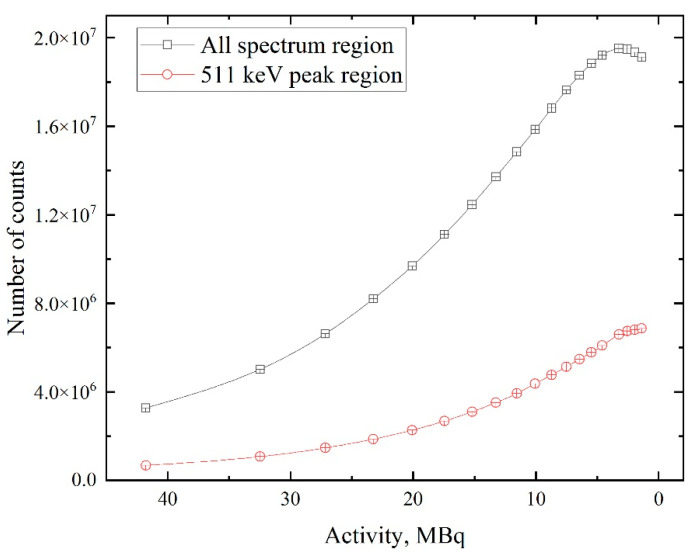
Dependence of the total number of counts in the whole spectrum of ^64^Cu isotope and in the range of positron annihilation line (511 keV) on the activity.

The presented data indicate that with decreasing source activity, a noticeable change in the annihilation peak occurs, both in terms of the shape of the spectrum (with increasing activity, the peak becomes wider) and in terms of overall statistics. This change is evident at the peak and background (see the logarithmic scale in Figure 4) parts of the spectra. This transformation likely affects the shape parameters of the DBS spectra. In particular, the number of counts increases with a decrease in activity. The dependence of the total number of events in the spectra on the source’s activity is presented in Figure 5.

**Figure 6:**
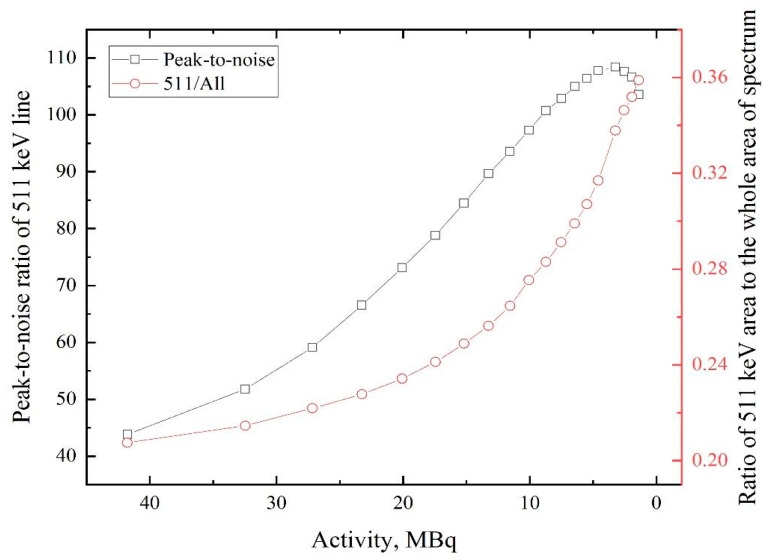
Dependences of 511 keV line peak-to-noise ratio and the ratio area under the line to the area of the whole spectrum on ^64^Cu isotope activity.

Figure 5 shows that an increase in the number of counts with activity drop characterizes not only the annihilation line but also the whole energy spectrum. This dependency is due to the increasing fraction of “good” signals and decreasing dead time ratio of the detector. A continuation of the experiment (at activity values lower than 1.3 MBq) is assumed to lead to a dramatic drop in intensity, as presented in Figure 5, due to the decay of radioactive ^64^Cu nuclei in the background of the highest effectiveness of the acquisition process. The start of this point is evident in Figure 5 (black line). The contribution of the 511 keV line in the whole spectrum and a peak-to-noise ratio of this line are characteristics that represent the spectrum quality. The dependences of these characteristics on the source’s activity are presented in Figure 6.

**Figure 7:**
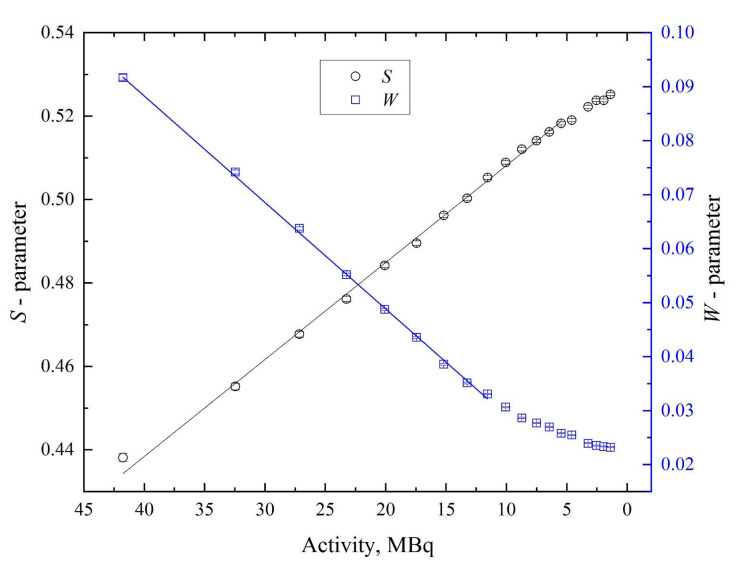
Dependence of *S*- and *W*-parameters of DBS on the activity of ^64^Cu source.

Despite the contribution of the 511 keV line increase with decreasing activity, the peak-to-noise ratio of this line rises until the activity is down to 2–5 MBq. The latter dependence correlates very well with the total number of counts (Figure 5) in the spectrum. The difference between the first and second curves in Figure 6 can be explained by the fact that when the annihilating peak starts to fade, other peaks that are attributed with ^64^Cu decay start to decrease (1022 keV, 1346 keV). This phenomenon will keep the ratio of 511 keV to all spectrum from decreasing quickly, which is not the case for the peak-to-noise ratio dependence. The dependence of *S*- and *W*-parameters on the activity of the obtained source for pristine samples is shown in Figure 7.

Figure 7 shows that the *S*-parameter increases and the *W*-parameter decreases with time. This effect is not due to changes in the defect structure of the material (one set of samples was used in the measurement) but rather to the dependence of the annihilation peak shape on the positron source activity. Based on the presented distributions (Figure 4), as the activity decreases, the peak becomes higher and narrower, which affects the *S*- and *W*-parameters.

**Figure 8:**
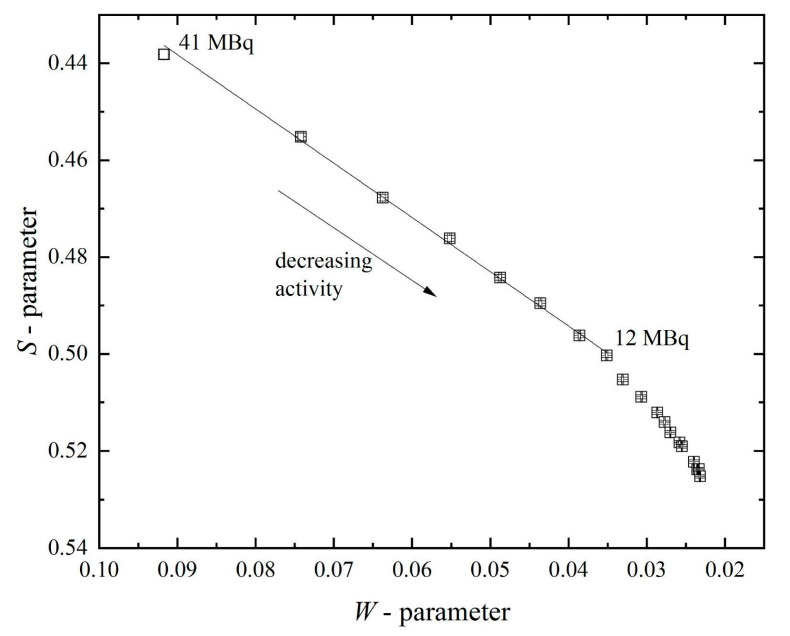
Dependence of *S*- on *W*-parameter for spectra with the different activity of positron source.

Another parameter, the *R*-parameter, helps evaluate the predominant type of defects present in the material. This parameter can be calculated as the ratio of the change in *S*-parameter to the change in *W*-parameter. Another way to evaluate the defect type is to determine the dependence of *S*- on *W*-parameters and to assess the change in slope. Such dependence is presented in Figure 8. The *R*-parameter is nearly constant at the activity region of 12–41 MBq (the slope is constant).

Despite the variable character of the shape parameters of the DBS spectra, the linearity in the concentration ranges of 7–41 MBq for *S*- and 12–41 for *W*- and *R*-parameters enables the experiment, and the obtained results can be compared quantitatively. The dependences of the *S*- and *W*-parameters become less linear when the source activity decreases to less than 7 MBq and 12 MBq, respectively. Still, the results of different measurements can be adequately compared by graphically overlaying the dependences of the shape parameters on the positron source activity.

**Figure 9:**
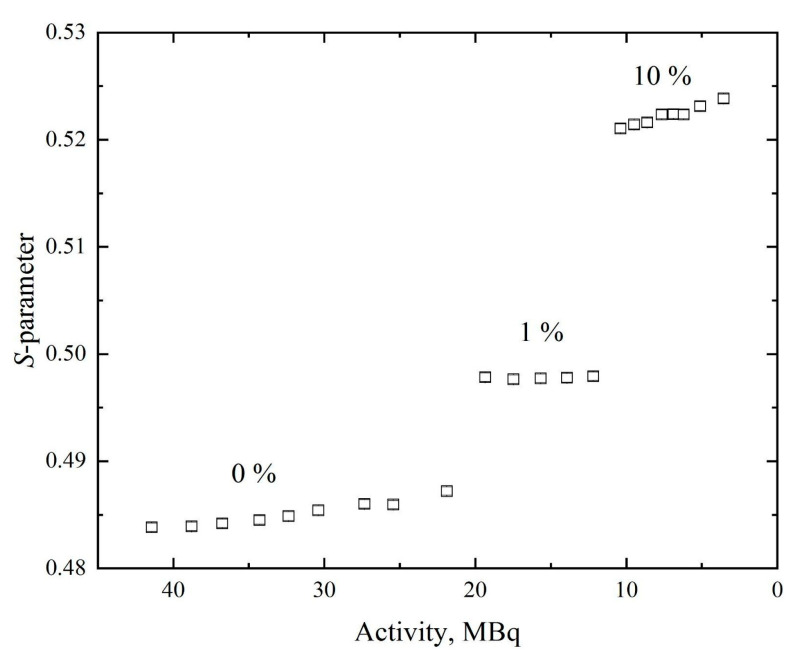
Dependence of *S*-parameter on activity for Ti samples with different dislocations density.

After assessing the dynamics of changes in the spectrometric characteristics of the positron source, its applicability to the study of defects was tested. Hence, samples with different concentrations of defects (dislocations) were prepared (see Section 2.2). For this experiment, 1 mg of copper foil was irradiated in 10^13^ n/cm^2^ flux of thermal neutrons for 3 h with a subsequent rest of 1 h out of the neutron field. The resulting activity of the source was approximately 42 MBq. The dependence of the *S*-parameter on activity value is presented in Figure 9.

Despite the change in *S*-parameter due to the rapidly changing source characteristics, the influence of changing the defect structure clearly has a greater effect on its value.

### 3.2. Performance of ^64^Cu at High Temperatures and in a Hydrogen Atmosphere

**Figure 10:**
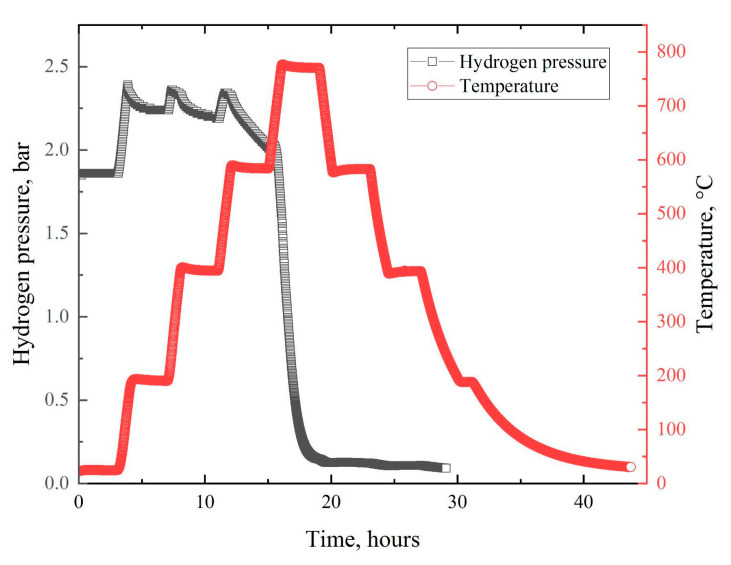
Temperature and hydrogen pressure profiles in dependence on time for heating of Ti samples with ^64^Cu positron source in vacuum and hydrogen atmosphere.

The profile of temperature in the vacuum chamber, as presented in Figure 10, was the same for the two experiments: heating in vacuum and heating in the hydrogen atmosphere. For the latter, hydrogen was pumped into the chamber to a pressure of about 1.8 bar.

From the presented hydrogen pressure profile, we can see areas of rapid growth associated with the heating stages. However, in the background of these peaks, starting from about 400 °C, the process of slow hydrogen sorption becomes noticeable. This is evidenced by the decreasing height of the pressure peaks associated with heating. In addition, the figure shows that the main sorption stage took place in the temperature range of 630–770 °C and lasted for approximately 2.5 h. Afterward (during the cooling stages), the sorption virtually ceased.

The absolute concentration of hydrogen in the titanium samples after saturation was 0.914 wt. % (around 30.7 at. %), and that in the copper source was 0.017 wt. %. The corresponding values for the virgin materials were less than 0.005 wt. % and 0.009 wt. %, respectively. Thus, copper did not accumulate hydrogen in this saturation mode, as expected.

**Figure 11:**
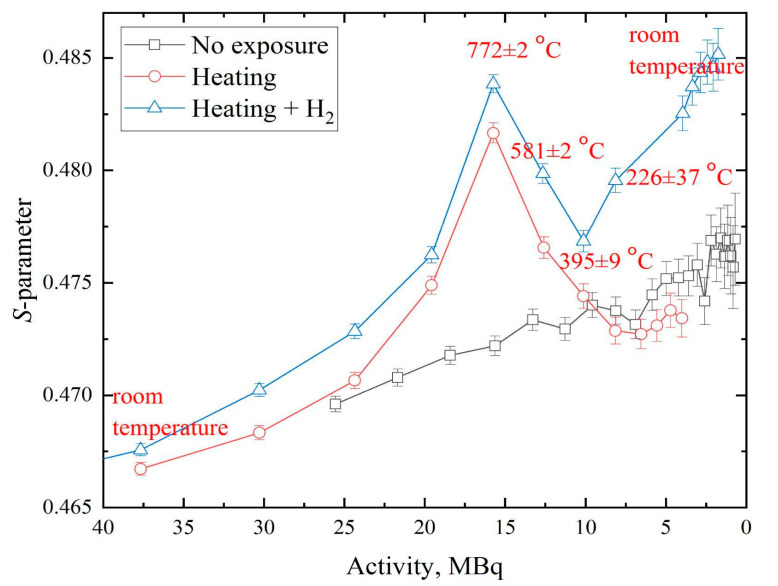
Dependence of *S*-parameter on activity of ^64^Cu source for Ti samples with no exposure, during heating, and hydrogen loading.

The dependences of the *S*-parameter on ^64^Cu activity during the heating of samples with source in the vacuum and hydrogen atmosphere are shown in Figure 11. The presented data show that the heating led to a faster increase of *S*-parameter than without heating. This effect was due to the increase of equilibrium vacancies concentration with temperature. At the beginning of the experiment with heating, the curve of the *S*-parameter aligned closely with the experiment without heating, confirming that the data from different (varying only in area) ^64^Cu-based positron sources agreed well.

Significant changes became noticeable when the sandwich was heated to 600 °C. We can conclude that the concentration of thermal vacancies exceeds the sensitivity threshold of this technique at 600 °C. This threshold could likely be lowered by using thinner foil (thus increasing the number of positrons annihilating in the studied sample). At the end of the heating experiment (when the temperature was again close to room temperature), the *S*-parameter was slightly below that of the initial material (without heating). The differences were small but appear to be due to the lower final concentration of defects in the sample, thanks to the longer temperature annealing. The changes in the W-parameter had a similar (but mirrored) effect and are not presented here.

In the hydrogen saturation experiment, the S-parameter curve was located above the vacuum heating curve, owing to the higher concentration of defects in the saturated samples. The small but stable difference in the defect concentration could be caused by both the difference in the initial state of the samples and the direct exposure to hydrogen (development of hydrogen-induced vacancies [6]). In general, the trends of the shape parameters of the DBS spectra when heated in a vacuum and in a hydrogen atmosphere are coincident in the temperature range 226–395 °C at the cooling stage. In this range, a sharp increase of the S-parameter was observed in comparison with vacuum annealing, during which the behavior of these parameters exhibited an opposite trend. This sharp increase in defectiveness of saturated samples is explained by phase transitions occurring during the experiment. According to the phase diagram presented in [36], the cooling of titanium with 30.7 at. % of hydrogen below 300 °C would start the formation of the delta hydride phase. Based on [3,37,38], this process leads to the growth of microstresses and dislocation density inside many materials, including Ti, and explains the sharp change in the trend of the line-shape parameters of DBS spectra.

## 4. Conclusions

This study is the first implementation of the ^64^Cu isotope for the DBS method to perform in situ measurements, taking into account all the features of obtaining and operating this source. The authors conclude that the positron source based on the ^64^Cu isotope is a prospective instrument for in situ defects investigation since it can be applied at high temperatures and in hydrogen media. However, the fast evolution of the source’s spectrometric characteristics should be taken into account.

The vital characteristics (line-shape parameters *S, W, R*) change in accordance with a simple linear model in the activity range of 12–40 MBq. This range is the most suitable for numerical comparison of different measurement data. However, the whole range of activity is suitable for the graphical comparison of the data or the analysis of line-shape parameter differences for a set of measurements.

Regarding the spectra quality and acquisition effectiveness, these characteristics improve with activity decrease down to 2–7 MBq.

The use of a copper-based isotope has shown to be effective in investigating thermal defects as well as hydrogen-induced defects in situ. The latter experiment demonstrated that the predominant process in the formation of hydrogen-induced defects when titanium was saturated to hydrogen concentration of about 0.9 wt% takes place during the cooling stage below 300 °C. This process relates to dislocations formation induced by hydride phase growth.

In the future, the authors of this work plan to use this technique to study promising hydrogen storage materials in situ processes of hydrogen thermodesorption as well as high-temperature sorption.

## Figures and Tables

**Table 1:** Main characteristics of HPGe detectors used for the DBS studies with ^64^Cu isotope.

	In-Air Experiments	In-Vacuum Experiments
Relative efficiency (%)	20	30
Resolution (full width at half maximum)	1.8 keV at 1.33 MeV, 0.85 keV at 122 keV	1.8 keV at 1.33 MeV, 0.875 keV at 122 keV
Peak/Compton	50:1	58:1
Crystal diameter (mm)	52	60.5
Crystal length (mm)	47	46.3
Distance from the window outside (mm)	6	6
Bias voltage (Vdc)	+3000	4000
Distance between detector and source (cm)	19	48

## Data Availability

The data presented in this study are available on request from the corresponding author. The data are not publicly available due to privacy reasons.
